# Live Tissue Imaging Shows Reef Corals Elevate pH under Their Calcifying Tissue Relative to Seawater

**DOI:** 10.1371/journal.pone.0020013

**Published:** 2011-05-27

**Authors:** Alexander Venn, Eric Tambutté, Michael Holcomb, Denis Allemand, Sylvie Tambutté

**Affiliations:** 1 Centre Scientifique de Monaco (CSM), Monaco, Principality of Monaco; 2 LEA CSM-CNRS ‘BIOSENSIB’, Monaco, Principality of Monaco; Northeastern University, United States of America

## Abstract

The threat posed to coral reefs by changes in seawater pH and carbonate chemistry (ocean acidification) raises the need for a better mechanistic understanding of physiological processes linked to coral calcification. Current models of coral calcification argue that corals elevate extracellular pH under their calcifying tissue relative to seawater to promote skeleton formation, but pH measurements taken from the calcifying tissue of living, intact corals have not been achieved to date. We performed live tissue imaging of the reef coral *Stylophora pistillata* to determine extracellular pH under the calcifying tissue and intracellular pH in calicoblastic cells. We worked with actively calcifying corals under flowing seawater and show that extracellular pH (pHe) under the calicoblastic epithelium is elevated by ∼0.5 and ∼0.2 pH units relative to the surrounding seawater in light and dark conditions respectively. By contrast, the intracellular pH (pHi) of the calicoblastic epithelium remains stable in the light and dark. Estimates of aragonite saturation states derived from our data indicate the elevation in subcalicoblastic pHe favour calcification and may thus be a critical step in the calcification process. However, the observed close association of the calicoblastic epithelium with the underlying crystals suggests that the calicoblastic cells influence the growth of the coral skeleton by other processes in addition to pHe modification. The procedure used in the current study provides a novel, tangible approach for future investigations into these processes and the impact of environmental change on the cellular mechanisms underpinning coral calcification.

## Introduction

Coral reefs are among the most biodiverse of marine ecosystems and hold significant ecological and economic value to tropical coastal nations worldwide [1]. Their existence depends on the calcification of scleractinian corals and other calcifying organisms to build and maintain reef structures, but surprisingly little is known about the cell physiology of coral calcification and other processes important to reef sustainability [2], [3]. Moreover, insights into fundamental aspects of coral physiology are needed for a clearer understanding of how coral reefs may respond to climate change and ocean acidification [4].

The physiology of the calcifying tissue of corals is one fundamental aspect of coral biology for which information remains sparse [5]. The cell layer overlying the coral skeleton, termed the calicoblastic epithelium, is involved in the extracellular production of the CaCO_3_ (aragonite) coral skeleton under its apical membrane [6]. To date, almost no physiological studies have been performed *in vivo* on the calicoblastic epithelium and many of its physiological properties remain undescribed [5]. This may be partly due to the fact it is difficult to access, as its apical side faces the massive CaCO_3_ skeleton and its basal side faces three other epithelia that separate it from the surrounding seawater (for a detailed histology of corals see [6]).

Determining how the calicoblastic epithelium alters the environment below its apical membrane is essential to understanding how coral biomineralization functions. For several decades it has been argued that corals increase the saturation state of aragonite at the tissue-skeleton interface to promote skeleton formation [7]–[10]. In addition to transporting calcium [11], the calicoblastic epithelium is proposed to elevate pHe on its apical side with respect to the surrounding seawater to achieve this increase in aragonite saturation state [9], [12]–[15]. However, the technical difficulties of accessing the calicoblastic epithelium have prevented investigations of subcalicoblastic pHe and other physico-chemical parameters in intact, living corals. Previous direct measurements have only been attempted invasively by insertion of a microelectrode via an opening cut through the tissue layers. [16]. Other estimates of pHe below the calcifying tissue of corals have been made indirectly in dead coral skeletons by geochemical methods to reconstruct the paleo-pH of seawater [17]–[20]. The application of non –invasive tissue imaging approaches to investigate pHe and other physico-chemical parameters at the coral tissue-skeleton interface would significantly facilitate physiological and geochemical research linked to coral calcification.

In addition to determination of pHe under the calicoblastic epithelium, measurements of pHi in the calicoblastic cells themselves are also required to attain a more detailed understanding of the cell biology underpinning calcification. Indeed, pHi modulates most aspects of cell function [21] and it is likely to play an important role in regulating enzyme activity and the speciation of dissolved inorganic carbon (DIC) transported from the surrounding seawater to the coral skeleton [22]. Our previous investigations into intracellular pH in corals have centred on isolated cells containing photosynthetic symbionts [23]. Determination of pHi in the calicoblastic epithelium and intact coral tissues in general has not been achieved to date.

In the current study we gained access to the calcifying tissue of the coral *S. pistillata* by working at the margin of corals grown on glass coverslips [24], where gaps between crystals allowed us to view the calicoblastic epithelium by inverted confocal microscopy. Coupled with fluorescent probes, this system provides a unique model for live-tissue imaging of both intracellular and extracellular physiological properties of the calcifying tissue. Working with carboxyseminaphthorhodafluor-1 (SNARF-1), we performed pH imaging of the calicoblastic epithelium of corals that were actively calcifying under flowing seawater in both light and dark conditions.

## Results

### Morphology of corals samples

**Figure 1:**
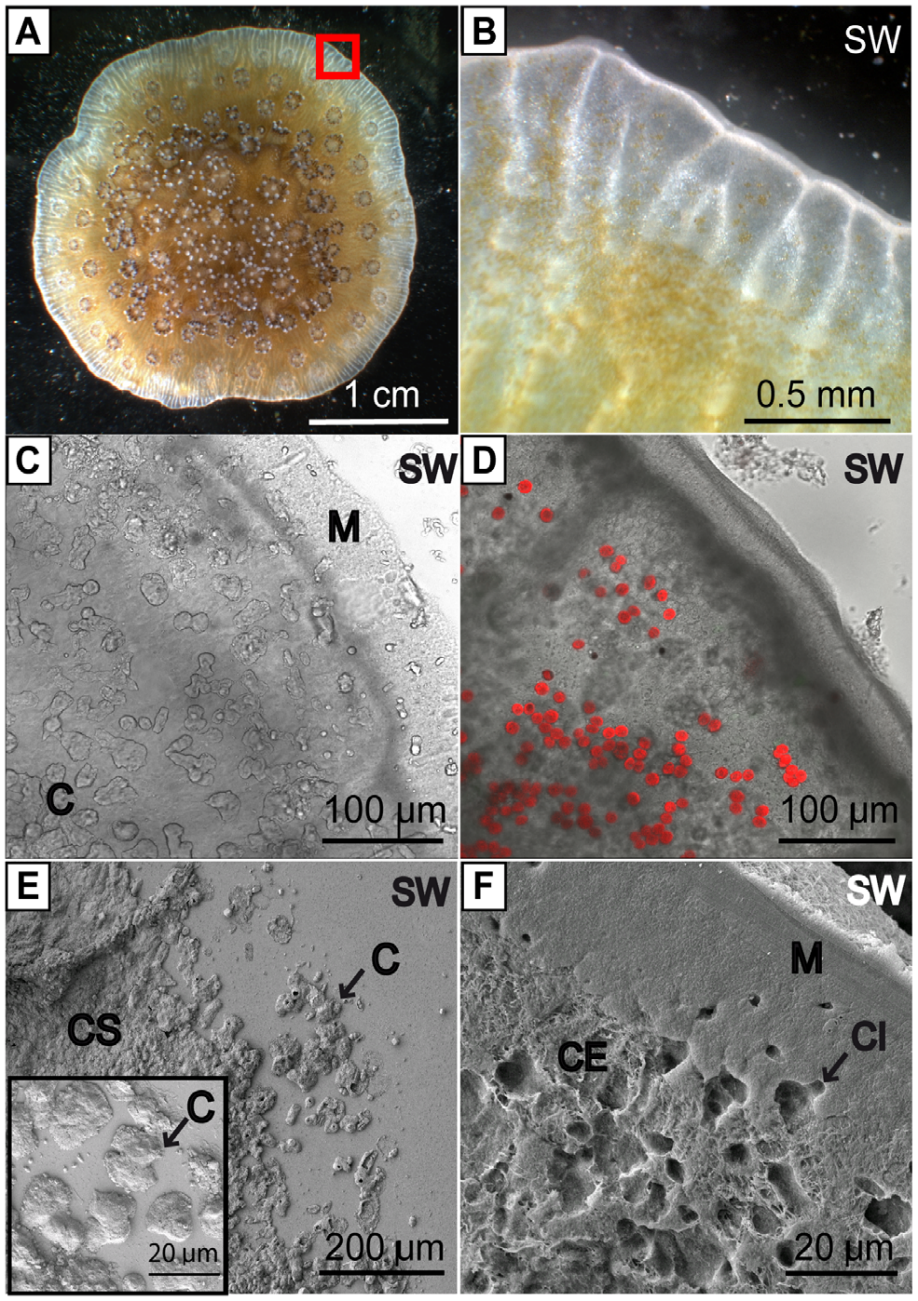
Morphology of *Stylophora pistillata* grown on glass coverslips. (A, B) Light microscope images taken from above, looking down on sample. (B) Zoom of red square indicated in (A). (C, D) *in vivo* transmitted light micrographs taken from below looking up through the coverslip, (C) to a focal plane just above the coverslip showing crystals under the coral tissue, and (D) at focal plane of 30 µm above the glass coverslip showing autofluorescence of symbiotic algae (red colour) (image merged with confocal micrograph). (E, F) FESEM images. (E) shows crystals on glass coverslip with tissue removed. Inset in (E) zoom on crystals. (F) shows the apical membrane of the calicoblastic epithelium (membrane usually facing the skeleton) with the crystals and coverslip removed. SW = position of exterior seawater. In (E) and (F) SW denotes position of seawater prior to fixation of the sample. M = distal margin of the sample. C = crystals. CS = Continuous skeleton. CI = impression left by crystal before removal of skeleton.

Samples of *Stylophora pistillata* grown out laterally across glass coverslips were viewed by light microscopy and field emission scanning electron microscopy [FESEM] to explore their morphology. Observations from above showed the distal margin of the samples were characterised by laterally extended tissue (Figure 1A,B). Inspection from below the samples by inverted light microscopy revealed that the tissue overlays calcium carbonate crystals (Figure 1C). When *in vivo* light images were obtained at a focal plane deeper in the overlying tissue, symbiotic algae residing in the endoderm cell layers were visible at 60 µm from the outer edge of the sample (Figure 1D) and free algae were seen moving rapidly within the coelenteron.

FESEM of samples with the tissue removed showed that isolated crystals and crystal bundles of various morphologies were joined to form a continuous sheet of skeleton at a distance that varied between 100 to 400 µm from the sample margin (Figure 1E). The term “crystals” is used to refer to all crystal types observed in the current study regardless of morphology. The morphology and growth of crystals in *S. pistillata* prepared on glass coverslips has been described previously [25].

Initial observations of the calicoblastic epithelium were performed on decalcified specimens removed from glass coverslips and analysed by FESEM (Figure 1F). Whilst the apical membrane of the calicoblastic epithelium presented a relatively smooth surface near the sample margin it formed a heterogeneous, uneven surface further into the sample. In certain areas, this heterogeneity clearly corresponded to impressions of isolated crystals that had been removed by the decalcification process. In other areas it was not possible to distinguish between impressions of the skeleton and spaces that may have been created by lifting of the calicoblastic epithelium away from the glass coverslip.

### *In vivo* imaging of the calicoblastic epithelium

**Figure 2:**
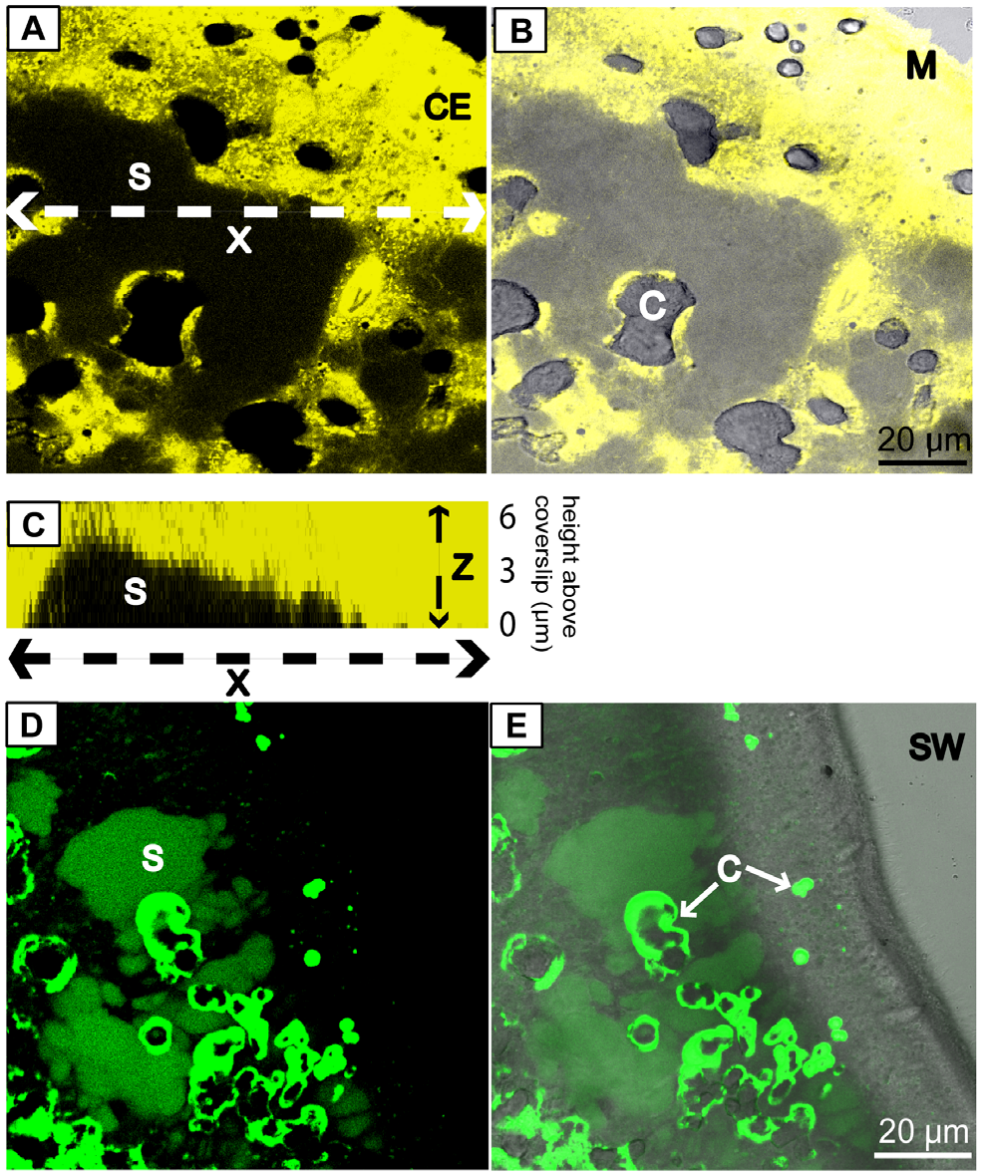
*In vivo* images of the calicoblastic epithelium of *S. pistillata*. (A) confocal optical section at 1 µm above the coverslip. Yellow = intracellular fluorescein diacetate (FDA) staining the calicoblastic epithelium. (B) Transmitted light image merged with (A) to show crystal detail. (C) Profile view showing vertical relief of (A) acquired by Z-stack reconstruction. (D) confocal optical section at 1 µm above the coverslip. Green = calcein in the extracellular subcalicoblastic medium and staining crystals. Crystals are stained more brightly than the subcalicoblastic medium. (E) Transmitted light image merged with (D) to show position of tissue. C = crystals. S = fluid-filled space under the calicoblastic epithelium. M = sample margin. CE = calicoblastic epithelium. X = position of Z-stack vertical reconstruction shown in (C).

Inverted confocal microscopy was performed on living samples stained with the cell-permeant dye fluorescein diacetate (FDA) (Figure 2A–C). Optical sections were captured from the level of the glass coverslip upwards into the tissue in order to image the calcifying tissue in both horizontal and vertical dimensions (Z-stack analysis). Transmitted light images were captured simultaneously to image the crystals and the gross tissue morphology (Figure 2B). Z- stacks revealed that the calicoblastic epithelium was generally in contact with the crystals and with the coverslip, but lifted away from the glass coverslip to heights of 2 to 10 µm in certain areas, creating spaces under the calicoblastic tissue (Figure 2C). These images revealed that the spaces were often larger than the underlying crystals. (Figure 2A–C).

To investigate extracellular spaces under the calicoblastic epithelium, samples were perfused with a solution of filtered seawater (FSW) containing cell-impermeable calcein. Analysis of z-stacks taken at the interface between the coral tissue and the glass coverslip showed that calcein stained the extracellular fluid under the calicoblastic epithelium (termed here subcalicoblastic medium) where the calicoblastic epithelium was lifted away from the coverslip (Figure 2D,E). By contrast calcein was not detectable in the calicoblastic epithelium itself. It remained under lifted areas of epithelium and did not occur as a continuous layer underneath the apical membrane. Calcein also brightly stained the skeleton and isolated crystals (Figure 2D,E). Crystals were observed to occur on the edges of areas of calicoblastic epithelium that were lifted away from the coverslip and under areas of tissue that were in close contact with the glass coverslip (Figure 2D,E).

### Analysis of calicoblastic epithelium pHe and pHi

**Figure 3:**
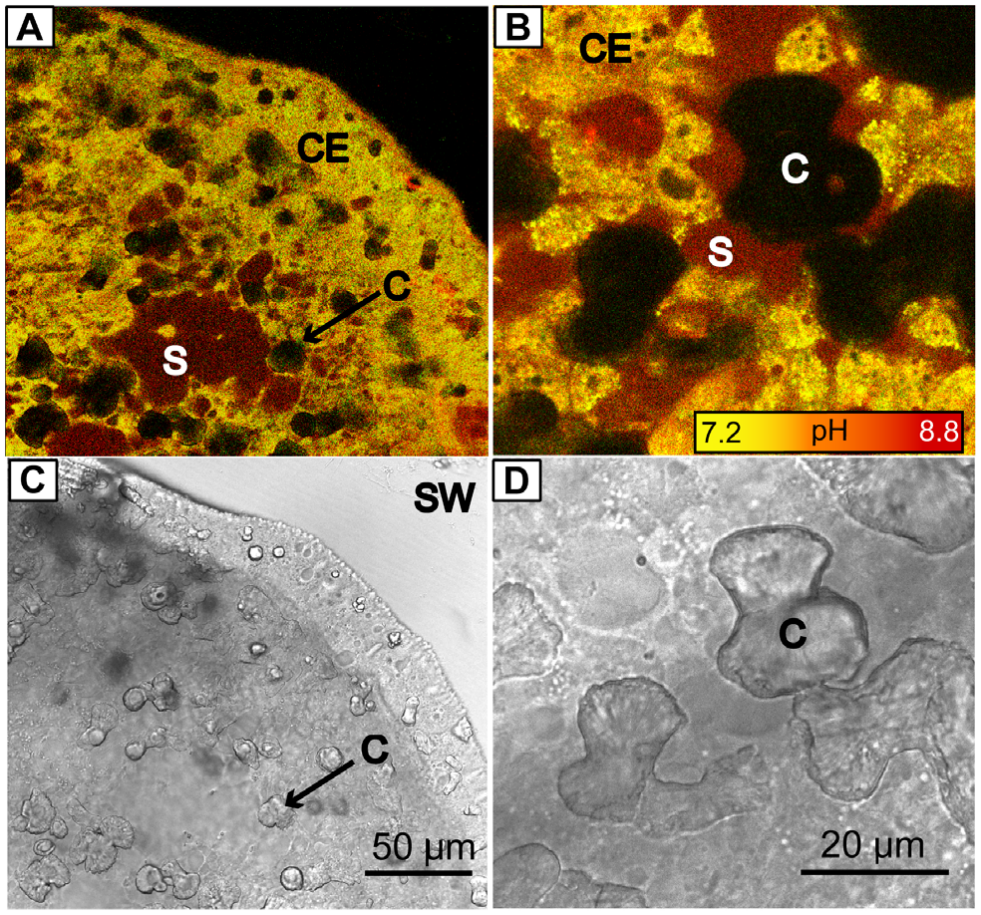
Distribution of pH in the calicoblastic epithelium of *S. pistillata*. Images obtained at 1 µm above the coverslip by inverted confocal microscopy. (A,C) 40× (B,D) 100× magnification. (A, B) combined images of SNARF-1 fluorescence obtained at 585 and 640±10 nm in samples stained with SNARF-1 AM and SNARF-1 simultaneously. Images (C, D) transmitted light images of the same area. C = crystal. S = fluid-filled space under the calicoblastic epithelium. CE = calicoblastic epithelium.

Imaging of pHi was performed in the calicoblastic epithelium by ratiometric analysis of SNARF-1 AM. Measurements of pHe were performed under areas of raised epithelium in the extracellular subcalicoblastic medium using SNARF-1. The calicoblastic tissue and subcalicoblastic medium were readily distinguishable due to differences in the fluorescence of the dye related to pH differences of the extracellular and intracellular compartments (Figure 3).

Calicoblastic epithelium pHi and subcalicoblastic medium pHe were determined in dark and light conditions. During dark and light experiments the pH of the seawater surrounding the coral sample was also measured by analysis of SNARF-1 at 100 µm and 2 mm from the sample. Calibration curves of intracellular and extracellular pH with SNARF-1 fluorescence are provided as Figure S1.

**Figure 4:**
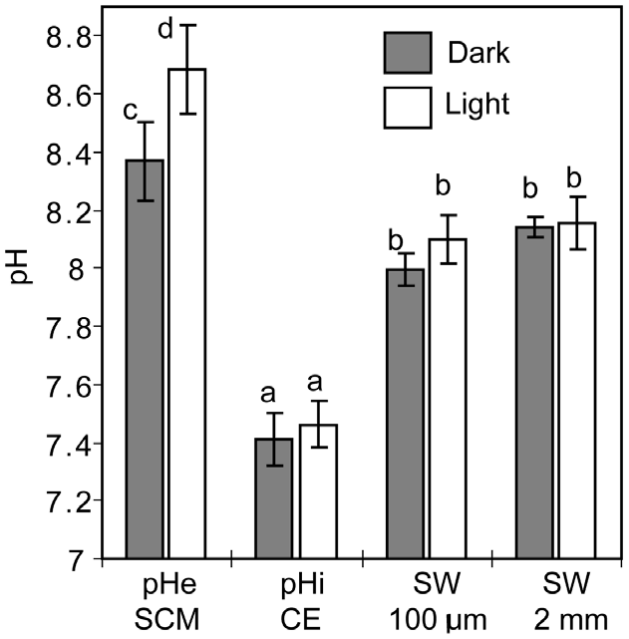
pH measurements in and under the calicoblastic epithelium of *S. pistillata*. Measurements obtained in the subcalicoblastic medium (SCM), the calicoblastic epithelium (CE) and in the surrounding seawater at 100 µm and 2 mm from the sample margin in light and dark conditions. Data are means ± s.d., n = 4 different colonies. Letters in superscript indicate homogenous subsets determined by Tukey's posthoc analysis.

The pH data were analysed by 2 way ANOVA with location of measurement (e.g. subcalicoblastic medium (SCM)) and light/dark treatment as factors. (2 way ANOVA, location F_3_,_24_ = 155.6, *P*<0.001; treatment F_1_,_24_ = 15.4, *P*<0.01, interaction F_3_,_24_ = 3.4, *P*<0.05.) As a significant interaction was found the data were then analysed by Tukey's posthoc analysis to identify significant differences between groups. Homogeneous subsets are shown in figure 4. Subcalicoblastic pHe in both light and dark conditions was significantly higher than pH in the surrounding seawater measured at both 100 µm and 2 mm from the sample. Subcalicoblastic pHe was significantly higher in light versus dark incubated samples. The mean values of seawater pH at 2 mm and 100 µm from the sample were not significantly different in light and dark conditions. Calicoblastic epithelium pHi remained unchanged between light and dark incubated samples (Figure 4).

### Association of the calicoblastic epithelium with growth of the underlying crystals

**Figure 5:**
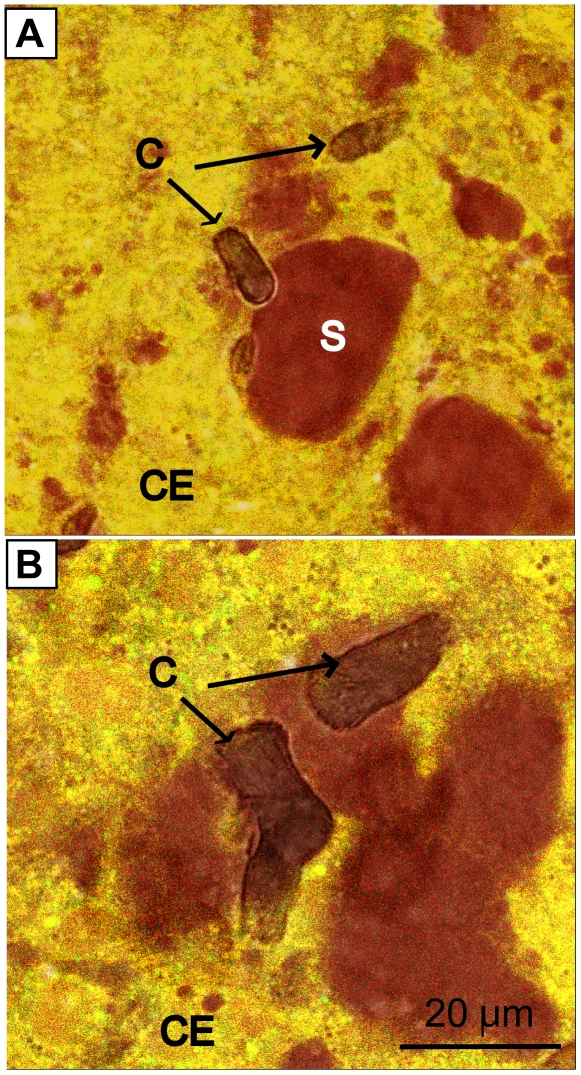
Spatial relationship of the calicoblastic epithelium and crystals of *S. pistillata*. Images obtained 1 µm above the coverslip by confocal microscopy on the same area of tissue before (A) and after (B) 5 h in aquarium conditions. Yellow = SNARF-1 AM in calicoblastic epithelium (CE). Red = SNARF-1 in subcalicoblastic medium (S). C = crystals.

Throughout the investigation crystals were consistently observed to be in contact with the calicoblastic epithelium, often at the periphery of spaces containing subcalicoblastic medium at elevated pH (Figure 2, 3, 5). Crystals were never observed to occur isolated in spaces containing subcalicoblastic medium, totally out of contact with the calicoblastic tissue. Similarly, when the growth of crystals was observed by imaging samples before and after periods of growth under aquarium conditions in the light, crystal growth appeared to occur in association with both calicoblastic tissue and the subcalicoblastic medium at the periphery of spaces created by lifted tissue (Figure 5).

## Discussion

Physiological data on the extracellular medium at the tissue-skeleton interface are rare for most multicellular calcifying organisms, in which access to the calcifying environment is frequently obstructed by the presence of multiple tissue layers and a shell or skeleton [5]. The current study overcame these issues by imaging the calcifying tissue at the margin of corals grown on glass coverslips by inverted confocal microscopy, where the incomplete skeleton allowed unobstructed access of the apical side of calicoblastic epithelium. As the *in vivo* imaging procedure described here allows physiological measurements to be made on living calicoblastic cells adjacent to calcium carbonate crystals, this approach could aid further studies on the cell biology of calcification.

The principal limitation of this approach is the uncertainty to whether observations of the calicoblastic epithelium made at the margin of coral samples grown on glass coverslips can be related to the epithelium over a fully-formed coral skeleton. There is however evidence that data acquired at margin of corals are relevant to understanding fundamental steps in the coral calcification process. Firstly, the current study and a previous investigation have established that calcium carbonate crystals were growing under the area of calicoblastic epithelium we investigated (Figure S2) [25]. This indicates that we were working with a functional epithelium specialized for calcification. Secondly, the morphology and arrangement of isolated crystals in our samples resemble crystals at the periphery of the basal skeleton disc of newly-settled juveniles of *Pocillopora damicornis* [26]. This suggests that the processes occurring under the growing margin of samples fixed to coverslips are linked to the primary steps of calcification [25].

### Extracellular pH under the calicoblastic epithelium

The chief result of the current study is that *S. pistillata* elevates pHe under the calicoblastic epithelium above pH of the surrounding seawater. pHe values were ∼0.5 and ∼0.2 pH units above seawater pH of 8.15 in light and dark conditions respectively. This finding provides experimental support to proposed mechanisms of calcification that argue that corals elevate pH under the calicoblastic epithelium at the tissue-skeleton interface in order to increase the concentration of CO_3_^2−^ in the total DIC pool, thus increasing the saturation state of aragonite and promoting skeleton formation [9], [12]–[14].

To verify if changes in subcalicoblastic pHe were controlled by internal mechanisms rather than shifts in pH in the external seawater we monitored pH in the seawater surrounding the corals during light and dark experiments. Several studies have demonstrated that corals are surrounded by a diffusive boundary layer (DBL) in which pH could be influenced by respiration and photosynthesis [27]–[29]. Under the perfusion flow rate used, mean differences in the pH of the seawater circulating in the perfusion chamber 2 mm and 100 µM from the margin of the coral were not significant in light and dark conditions, suggesting pH shifts in the surrounding seawater were not responsible for the elevation of pHe observed under the calicoblastic epithelium.

Subcalicoblastic pHe elevation may have occurred by the removal of protons via an active uptake mechanism on the apical membrane of the calicoblastic epithelium [9], [12], [16]. Supporting evidence includes the characterisation of a Ca^2+^-ATPase in the coral *Galaxea fascularis* by a pharmacological approach [30] and the localization of a Ca^2+^-ATPase in the calicoblastic epithelium of *S. pistillata* [31], that is proposed to remove protons from the subcalicoblastic medium. It has been suggested that coral Ca^2+^-ATPase activity is higher in the light as a result of greater ATP availability due to higher rates of respiration stimulated by algal photosynthesis [16]. This may account for increases in subcalicoblastic pHe in light conditions observed in the current study and inferred from geochemical studies [14] and work with microelectrodes [16].

The pHe data obtained in the current study correspond with several studies that have estimated pHe at the site of calcification using geochemical evidence in coral skeletons [14], [32], [33]. Indeed certain geochemical studies report boron isotope signatures in field-collected corals that indicate a range in subcalicoblastic pHe almost identical to that obtained in the current study e.g. pHe 8.3 to 8.8 [32]. The similarity of these estimations derived from isotope analysis to our current pHe values further supports the interpretation that our values obtained under the calicoblastic epithelium are relevant to understanding the physico-chemical conditions under which coral skeletons are formed. Furthermore from the geochemical perspective, our procedure of taking *in vivo* measurements in the subcalicoblastic environment may help validate indirect pH estimates made in dead coral skeletons by isotope analysis. Much further work is needed to perform this validation, including a wider characterisation of physico-chemical parameters below the calicoblastic epithelium, over a wider range of environmental conditions and in other species of coral. This may require performing *in vivo* analysis by confocal microscopy and geochemical analysis of coral skeletons in a tandem study.

### Estimates of aragonite saturation state derived from subcalicoblastic pHe measurements

To investigate if the values of pHe in the subcalicoblastic medium obtained in the current study could translate to aragonite saturation states that would favour calcification, equilibrium calculations performed with the software CO2Sys [34]. To do this, we integrated our pHe data with a number of assumed values for carbonate chemistry parameters. Carbonate chemistry was calculated using carbonate constants of [35] as refit by [36] and [37] for sulfate. For simplicity, processes likely to be important in coral calcification such as active calcium transport and the addition of organic molecules were not considered.

Seawater is assumed to enter the coral with the following values of salinity 38 , temperature 25°C, nutrients 0, DIC 2177 µmol/kg, pH_NBS_ 8.15, alkalinity 2500 µmol/kg, and pass via a paracellular pathway into the subcalicoblastic medium. We assume that during transit, respiratory DIC diffuses from the cells into the seawater, elevating total DIC in the subcalicoblastic medium. A 50% dilution of seawater DIC by respiratory DIC is assumed, based on the middle of a range of estimates of the contribution of respiratory CO_2_ to skeletal carbonate ∼10% [38] to 74% [39]. DIC is assumed to be constant in light and dark conditions at 4354 µmol/kg in the subcalicoblastic medium. Lastly, we assumed that pHe values measured in the current study were achieved by proton removal as the primary mechanism for elevating pHe [and thus alkalinity]. Based on these criteria, Table 1 provides estimates of aragonite saturation state below the calicoblastic epithelium in both light and dark conditions. Comparison to previous research involving inorganic studies of calcium carbonate crystal formation indicate that the aragonite saturation states derived from our pHe measurements are potentially favourable to aragonite crystal growth in both light and dark conditions [10].

**Table 1:** Estimates of aragonite saturation state in the subcalicoblastic medium.

Treatment	pH (NBS)	pCO_2_ µatm	Alkalinity µmol/kg	Aragonite saturation state
Light	8.69	210	5869	19.9
Dark	8.36	532	5196	11.0

Aragonite saturation states were derived from pHe estimates made in the current study and assumed carbonate chemistry parameters (see discussion text for details).

### Intracellular pH of the calicoblastic epithelium

The values we obtained in the calicoblastic epithelium (pH 7.4±0.09) are close to values we obtained in isolated *S. pistillata* endoderm cells [23]. These data confirm the proposal made previously based on pHi in cell isolates that the intracellular environment of the calicoblastic epithelium favours HCO_3_^−^ as the dominate form of DIC, thus it is unlikely that the calicoblastic cell secrete CO_3_^2−^ as part of the calcification process [23], [40]. Conversion of DIC to CO_3_^2−^ must occur extracellularly in the comparatively high pH subcalicoblastic medium. This strategy contrasts with recent findings on certain unicellular benthic calcifiers, notably hyaline foraminifera, which convert DIC to CO_3_^2−^ intracellularly by elevating pH in aggregations of seawater vacuoles that are delivered to the site of calcite production [41], [42].

It is important to note that although calicoblastic epithelium is proposed to remove protons from the subcalicoblastic medium by a Ca^2+^ ATPase, no change in pHi was observed between light and dark conditions. The regulatory mechanisms of pH that account for this stability in calicoblastic epithelium pHi are undescribed in corals but may involve cation or anion exchangers and/or transporters on the basal side of the epithelium. Previous work has proposed that protons taken up by the calicoblastic epithelium are transported by an unknown mechanism to the coelenteron, where they are neutralized by OH^−^ ions produced by photosynthesis [13], [43].

### Association of the calicoblastic epithelium and underlying crystals

Observations of calicoblastic epithelium revealed that there are zones in which the calicoblastic epithelium is lifted creating spaces under the tissue and other zones where it is in close association with the underlying crystals. Looking at the literature both these morphologies have been described by structural studies of the calicoblastic epithelium. Certain studies have described a space of greater than 1 µm between the tissue and skeleton [6], [44], [45] in which the skeleton is proposed to precipitate [7], [46]. Other studies have described a tight association between skeletal crystals and the overlying epithelium [47], [48]. An interesting observation is that although subcalicoblastic compartments in the present study were relatively high pHe and potentially favourable to the precipitation of aragonite [Table 1], crystals were never observed to occur in the middle of subcalicoblastic compartments alone, out of close contact with the calicoblastic epithelium. Additionally, confocal optical sections consistently revealed that at least a part of each crystal was in close contact with the adjacent tissue. Further *in vivo* investigations are required to better characterise the interface between tissue and crystal, ideally at the nanometric scale, taking care to consider the optical complexities of working with living tissue [49]. Nevertheless, the data currently available from our study suggest that in addition to increases in subcalicoblastic pHe, crystal growth may also be promoted by other factors which require the close contact of the calicoblastic epithelium. Whether these factors are modifications of the physicochemical environment by ion transport and enzymes on the surface of the apical membrane (e.g. carbonic anhydrase [43]) or biochemical modifications by an organic matrix remains unknown. Clearly, much further work is necessary to understand the events leading to crystal growth under the calicoblastic epithelium of corals.

### Conclusions and future work

In conclusion, we report direct measurement of pHe under the coral calicoblastic epithelium that demonstrate that pHe is elevated below the calicoblastic apical membrane relative to the surrounding seawater. The pHe values reported here are consistent with a range of literature data including estimates made by boron isotope analysis of coral skeletons. We also report the first measurements of pHi in the calicoblastic epithelium.

Aside from the work needed to better understand the mechanism of calcification, the current work will also benefit studies that investigate how pHe, pHi and other physiological factors are impacted by environmental change. For example it is not currently known how well corals are able to buffer pH in the calcifying tissue under changes in external seawater pH and carbonate chemistry anticipated to occur under projected scenarios of ocean acidification. If changes in pHi or pHe are identified, they potentially have a role in shaping the response of calcification processes. Exploring these issues and investigating the links between pH regulation and the rates of calcification will be an important contribution to the wider understanding of the susceptibility of reef corals to global climate change.

## Materials and Methods

### Preparation and maintenance of corals

All experiments were conducted on samples prepared from colonies of *Stylophora pistillata* maintained at the Centre Scientifique de Monaco. Samples were prepared by the lateral skeleton preparative assay [24], [25]. Briefly, microcolonies of *S. pistillata* were allowed to rest on glass slides so that, the basal portion of the colony grew out over the slide as a thin sheet. Pieces of sheets were sectioned from the colonies with a razor blade and fixed with resin (Devcon™) to circular glass coverslips (Figure 1). Pieces of corals were then left to grow out across glass coverslips in aquariums supplied with flowing seawater from the Mediterranean sea (exchange rate 2% h -1) at a salinity of 38, under irradiance of 170 µmol photons m^−2^ s^−1^ on a 12 h: 12 h photoperiod. Algae were periodically removed from the glass coverslip by a razorblade.

### Experimental conditions during microscopy

Microscope observations and pH analysis of coral samples were performed under a constant perfusion of flowing filtered seawater (FSW) on a temperature controlled microscope stage (Temperable Insert P, PeCon) maintained at 25°C. The pH of the FSW was pH 8.1±0.05 (mean and range, electrode calibrated on NBS scale). Periodic checks of total scale pH measurements (made with m-cresol purple) in the aquarium from which SW was drawn gave values of 7.97±0.04 (mean and range).

A series of tests were performed to optimise the experimental set up. First, the perfusion rate was optimised to ensure that pH measurements were conducted in samples that experienced stable oxygen concentrations in the surrounding seawater. Oxygen concentration was measured by placing a needle type oxygen microsensor (PreSens) in the seawater 2 mm from the sample and adjusting the perfusion rate until oxygen levels remained stable at 260±20 µM in the perfusion chamber under both light and dark conditions. Typically this required a 50%/min renewal rate of a 2.5 ml volume of seawater for a coral sample of 1 cm^2^.

Second, tests were performed to confirm samples were calcifying under the selected levels of temperature and flow. Samples were incubated in a seawater solution of 160 µM calcein for 5 min to mark the boundary of crystals under the coral tissue. After removal of calcein from the surrounding seawater, corals were then placed under the conditions of perfusion described above. Inspection of samples by confocal and light microscopy confirmed formation of new crystals and growth of existing crystals whilst under perfusion on the confocal microscope under both light and dark conditions (Figure S2).

### Dye loading

The intracellular dyes FDA and SNARF-1 AM were prepared from stock solutions in DMSO, with the final concentration of DMSO no greater than 0.1% v/v and were added to samples at 25 µM and 10 µM respectively. SNARF-1 AM solutions also contained 0.01% Pluoronic acid. Samples were incubated in dyes for 15 min, before washing by perfusion with FSW. Solutions of extracellular dyes, calcein and SNARF-1, were prepared in FSW at 40 and 50 µM respectively and were added to samples by perfusion for 15 min. Samples were then washed by perfusion with FSW. Calcein and SNARF-1 were detectable in the subcalicoblastic medium for 10 min after rinsing and placing the coral under FSW perfusion. SNARF-1 and SNARF-1 AM were purchased from Invitrogen. Calcein and FDA were purchased from Sigma-Alrich.

### *In vivo* imaging of corals

Macroscopic images of corals were acquired using a Leica Z16APO (Leica™ Microsystems) under white light. Confocal microscopy was performed with a Leica SP5 confocal laser scanning microscope with the pin hole set at 1 Airy Unit using a ×40 or ×100-fold oil immersion lens. Transmitted light images were captured simultaneously with confocal optical sections in a separate channel. Confocal microscopy of samples was conducted from below. Samples were analysed by Z-stack analysis by capturing optical sections starting from the focal plane at the base of the crystals on the glass coverslip moving upwards to a maximum of 10 µm into the coral tissue in 0.5 µm steps.

Coral samples stained with FDA or calcein were imaged using excitation at 488 nm with emission capture 520±10 nm. Extracellular SNARF-1 and SNARF-1 AM were excited at 543 nm and fluorescence emission was captured in two channels 585 and 640±10 nm. Autofluoresence of algal chlorophyll was captured at 690±10 nm.

Non-stained samples were examined for interference by fluorescent proteins. Whilst green fluorescent proteins are sometime visible in samples of *S. pistillata*, fluorescent proteins were not detected in the calicoblastic epithelium when excited at 488 nm or 543 nm.

### Field emission scanning electron microscopy

FESEM was performed using a JEOL JSM 6700F field emission scanning electron microscope Centre Commun de Microscop Appliquée at the University of Nice Sophia-Antipolis. Preparation of samples, including removal of tissue or skeleton, and FESEM observation were carried out as described by [48].

### Determination of pHi and pHe

pHi and pHe were determined by ratiometric analysis of SNARF-1 [50]. Intracellular pH (calicoblastic epithelium) and extracellular pH [subcalicoblastic epithelium] were determined using two separate calibrations. Calibration of intracellular pH was performed *in vivo* on isolated *S. pistillata* cells between pH 6 and 8.5 as described previously [23]. Extracellular pH calibration was performed by determining the ratio of SNARF-1 fluorescence in FSW containing 50 µM SNARF-1 adjusted to the range of pH 7–9 (NBS scale) (Figure S1) [41]. Both calibrations were performed in light and dark conditions (Figure S1) In each calibration the 585/640 nm SNARF-1 fluorescence intensity ratio (R) was related to pH by the following equation:
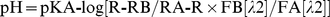
where F is fluorescence intensity measured at 640 nm (*λ*2) and the A and B represent the limiting values at the acidic and basic end points of the titration respectively. Ratio (R) was measured in digital regions of interest [ROIs] created in the subcalicoblastic medium and in the calicoblastic epithelium. Using this approach pH was determined in samples stained simultaneously with both SNARF-1 AM (for pHi) and SNARF-1 (for pHe) (e.g. figure 3) or each dye separately.

Background fluorescence values were recorded prior to loading with dye, and subtracted from the measurements used for pH calculation. Short Z-stacks (5 µm) were performed for each pH measurement to check that pH was stable through the area of epithelium or subcalicoblastic medium being measured (Fig. S1b).

### Measurement of pH in light and dark conditions

Samples were treated to darkness or light-levels that matched the conditions under which they were cultured (170 µmol photons m^−2^ s^−1^ PAR). Light was provided by a fibre optic light source (Bioblock Scientific, SFBH Mechanik GmbH). Following 15 min dark or light loading of SNARF-1 AM and SNARF-1 as described above (dye loading), ten measurements of pHi and pHe were taken during 20 min under perfusion to check pH values were stable. Samples were perfused with 50 µM SNARF-1 in seawater and pH was measured in the seawater in the light or dark at 100 µM and 2 mm from the edge of the sample during the experiment. Total exposure time of corals to SNARF-1 or SNARF-1 AM did not exceed 40 min post loading. During this time polyps of colonies remained expanded and visibly healthy. The viability of coral cells exposed to SNARF-1 in light and dark conditions has been assessed previously [23].

## Supporting Information

Figure S1 **Calibration of intracellular and extracellular SNARF-1.** (A) Calibration of intracellular (pHi) and extracellular (pHe) pH with the ratio of SNARF-1 fluorescence at 585 and 640 nm. See methods for details. (B) The stability of pHi in the calicoblastic epithelium (CE) and pHe in the subcalicoblastic medium (SCM) at different heights above the coverslip obtained by Z-stack analysis.
(TIF)

Figure S2 **Crystal growth in coral samples under experimental conditions used in the study.** Confocal images merged with transmitted light images of crystals under the calicoblastic epithelium following a short incubation with calcein at time zero (T = 0 h) and then after 5 hours (T = 5 h) under seawater perfusion in place on the confocal microscope.
(TIF)
