# Supplementary figures and images for: Live Tissue Imaging Shows Reef Corals Elevate pH under Their Calcifying Tissue Relative to Seawater
Source: PLoS One. 2011 May 27;6(5):e20013. doi: 10.1371/journal.pone.0020013 (PMC3103511; doi:10.1371/journal.pone.0020013)

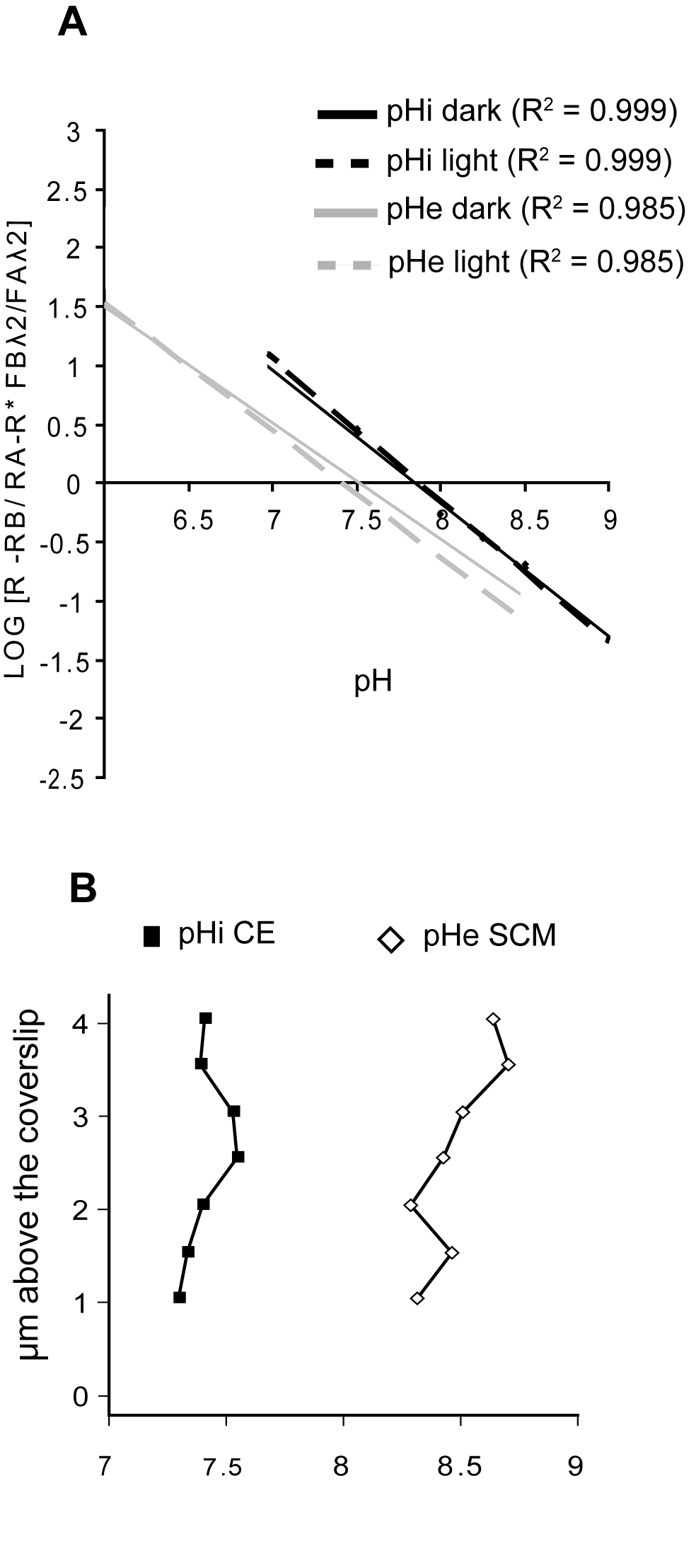

Supplement: Figure S1 — Calibration of intracellular and extracellular SNARF-1. (A) Calibration of intracellular (pHi) and extracellular (pHe) pH with the ratio of SNARF-1 fluorescence at 585 and 640 nm. See methods for details. (B) The stability of pHi in the calicoblastic epithelium (CE) and pHe in the subcalicoblastic medium (SCM) at different heights above the coverslip obtained by Z-stack analysis. (TIF) [file pone.0020013.s001.tif]

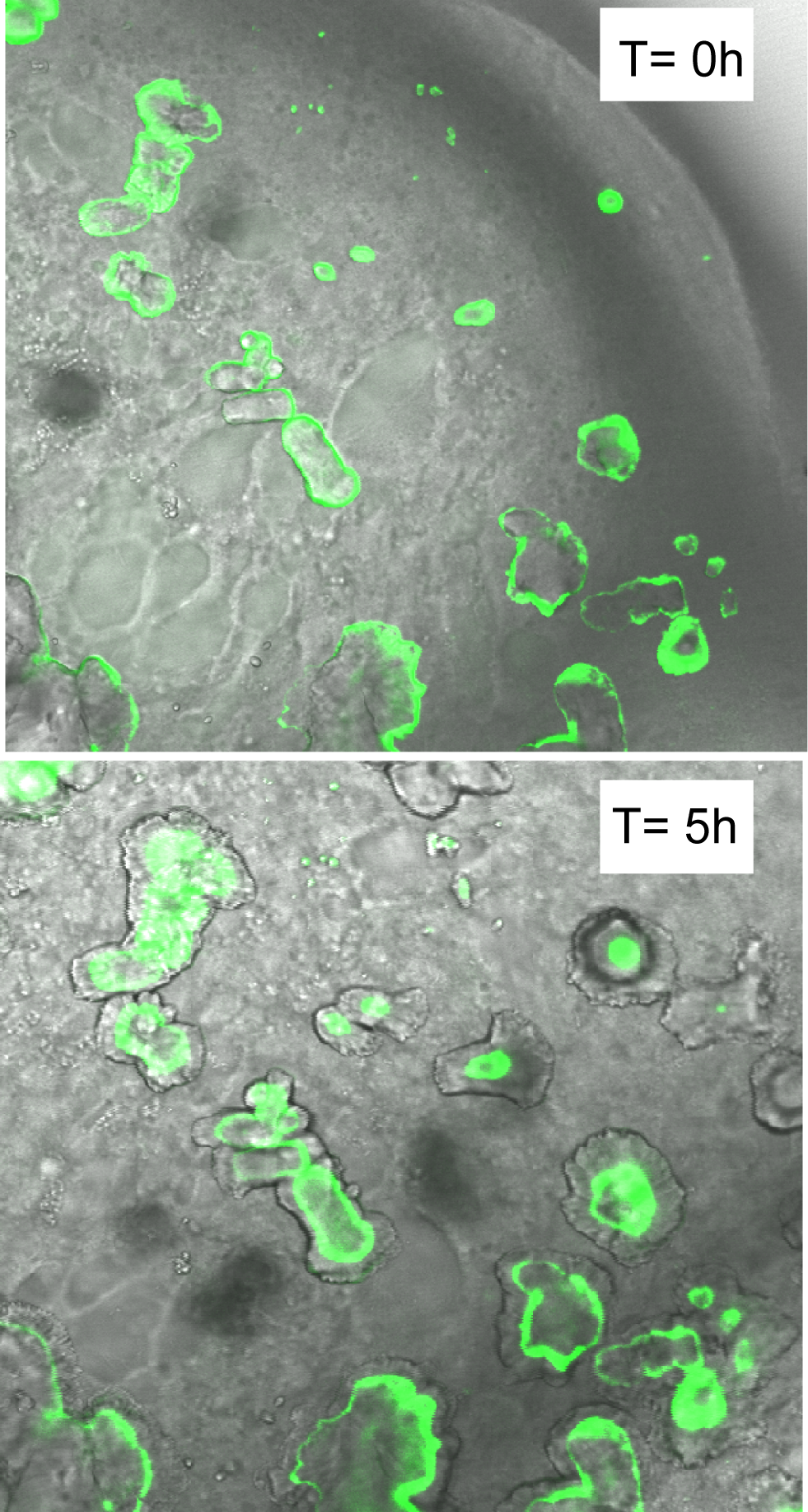

Supplement: Figure S2 — Crystal growth in coral samples under experimental conditions used in the study. Confocal images merged with transmitted light images of crystals under the calicoblastic epithelium following a short incubation with calcein at time zero (T = 0 h) and then after 5 hours (T = 5 h) under seawater perfusion in place on the confocal microscope. (TIF) [file pone.0020013.s002.tif]
